# The evaluation of the effect of tafluprost on the intraocular pressure of healthy male guinea pigs under different light‐and‐darkness regimes

**DOI:** 10.1002/vms3.1082

**Published:** 2023-02-09

**Authors:** Arghavan Armin, Farnoosh Arfaee, Saeed Ozmaie, Ahmad Asghari

**Affiliations:** ^1^ Department of Clinical Sciences, Faculty of Specialized Veterinary Sciences The Science and Research Branch of Islamic Azad University Tehran Iran

**Keywords:** glaucoma, guinea pig, intraocular pressure, light, ocular hypertension, tafluprost

## Abstract

**Background:**

Ocular hypertension is one of the most underdiagnosed ocular abnormalities among guinea pigs around the world.

**Objectives:**

The current study investigates the effect of 0.0015% preservative‐free tafluprost ophthalmic solution (Zioptan) on the intraocular pressure of 16 healthy male guinea pigs (*Cavia porcellus*) under different light/darkness regimes.

**Methods:**

All guinea pigs received a single drop of tafluprost at 5:30 in the right eye, whereas the contralateral eyes served as control to receive a placebo. Then, the animals were randomly divided into two groups; group A was exposed to light, whereas group B was placed in darkness from 5:30 to 18:00. Rebound tonometry (TonoVet) was instrumented to measure IOP values at 5:30 (baseline), 6:00, 7:00, 8:00, 9:00 and then every 3 h until 18:00.

**Results:**

The maximum IOP reduction associated with tafluprost was observed at 6:00 by −1.4 ± 1.1 mmHg (*p*‐value = 0.026) and −2.5 ± 1.2 mmHg (*p*‐value = 0.011) in group A and B, respectively (repeated measure ANOVA test). There was a significant difference between the mean right and left eye IOP values in both groups at 5:30, 6:00, 7:00 and 8:00 (*p*‐value <0.05), which was greater in amount in group B compared to group A due to the effect of darkness on IOP reduction.

**Conclusions:**

It is suggested that the variations of IOP in different light/dark conditions be taken into consideration when applying ocular hypotensive agents on guinea pigs’ eyes.

## Graphical abstract

The experiments investigates the effect of 0.0015% preservative‐free tafluprost ophthalmic solution (Zioptan^®^) on the intraocular pressure of 16 healthy male guinea pigs (Cavia porcellus) in group A and B, which were exposed to light and darkness for 12 hours, respectively. Rebound tonometry (Tono‐Vet^®^) was instrumented to measure IOP values at 5:30 (baseline), 6:00, 7:00, 8:00, 9:00 and then every 3 hours until 18:00. There was a significant difference between the mean right and left eye IOP values in both groups at 5:30, 6:00, 7:00 and 8:00 (*p*‐value < 0.05), which was greater in amount in group B compared to group A due to the effect of darkness on IOP reduction.

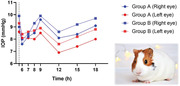

## 1 INTRODUCTION

Glaucoma is one of the driving causes of visual deficiency around the world. Due to the progressive nature of the ailment and the difficulty of ocular disease diagnosis in animal species, timely diagnosis and proper treatment are necessary for vision loss prevention (Komáromy et al., 2019; Weinreb et al., 2014). Although guinea pigs are widely kept as pet companions and laboratory animals, there have been a few investigations on the prevalence of ocular diseases in this specie (Williams & Sullivan, 2010).

One such study reveals that in a population of 1000 guinea pigs without any apparent changes in health and behaviour, 45% of them were diagnosed with ocular conditions associated with glaucoma, namely cataracts (21%) and lens lesions (17%) (Williams & Sullivan, 2010). These animals often conceal their disease symptoms instinctively in the wild being a prey specie not to steer the attention of predator species (Edis & Pellett, 2018). As a result, several chronic diseases of this specie may remain underdiagnosed and progress into advanced stages at the time of veterinary visits, such as prolonged ocular hypertension as a major glaucoma risk factor (Komáromy et al., 2019).

The reduction of the IOP by topical ophthalmic solutions is currently the mainstay of glaucoma therapy at the initial stages of the disease (Ruangvaravate et al., 2020). However, the interspecies differences among mammalian species in aqueous humour production and drainage pathways, as well as metabolic and anatomical differences, are responsible for the different efficacy of ocular hypotensive medications (Ofri, 2002; Papadia et al., 2011).

Moreover, it is believed that the daily cycles of light and darkness among other environmental factors can influence the diurnal pattern of IOP fluctuations (Buijs & Kalsbeek, 2001; Cahill & Menaker, 1989). In this study, we aim to evaluate the effect of tafluprost as a novel ocular hypotensive solution regarding the effect of light and darkness on IOP in the guinea pig.

## 2 MATERIALS AND METHODS

To evaluate the IOP‐lowering effect of tafluprost, a total of 16 healthy adult American Pigmented male guinea pigs (*Cavia Porcellus*) were obtained for this study. All animals were 18‐month old and weighed 550 g on average at the beginning of the experiment. To confirm ocular health prior to the study, a complete ophthalmologic examination was performed on each animal, which involved fluorescein staining (Fluorescein Glostrips, Nomax Inc, St. Louis, USA), Schirmer tear test (Schirmer‐Tranentest Vet, Eickemeyer, Tuttlingen, Germany), rebound tonometry (TonoVet, iCare; Helsinki, Finland), slit‐lamp biomicroscopy (Kowa SL‐15; Kowa, Tokyo, Japan), direct (WA11710 Ophthalmoscope, Welch Allyn Inc, NY, USA) and indirect ophthalmoscopy (Binocular Indirect Ophthalmoscope, Welch Allyn Inc, NY, USA).

Two weeks before the study, all animals were placed in a laboratory room with no windows to recover from shipping‐related stress and to acclimatize to the new light/darkness regime, which consisted of 12 h of light followed by 12 h of darkness (Devlin & Kay, 2001; Reppert & Weaver, 2002). The application of light was from above the animals’ heads at all times during the light phase provided by a white 40‐W LED lamp accompanied by a yellow 9‐W LED lamp. To avoid any IOP irregularities that would affect the study results, all IOP measurements during the dark phase were performed in the same room using dim red light provided from the room corner (Aihara et al., 2003; Liu et al., 1999). A same diet consisting of fresh fruits and vegetables and guinea pig commercial pallets was provided for all animals as nutrition is believed to influence IOP due to its effect on metabolism (Green et al., 2008). There was no food restriction for the animals, and water was ad liberum at all times before and during the experiment. White pine shavings were used to cover the floor of individual boxes (0.5 m × 0.5 m), which housed each animal. The animals were kept for 4 weeks in a laboratory room (3 m × 4 m) with a constant temperature of 25°C with no windows to prevent natural light entry.

To study the IOP‐lowering effect of tafluprost in different light/dark conditions, all guinea pigs were randomly divided into two groups (A and B), each consisting of eight animals. All guinea pigs were conscious during the experiments, and no topical anaesthetics were used due to the effect of anaesthetic drugs on the ocular blood flow and IOP (Blumberg et al., 2007). To avoid any excitement, nervousness and excessive movements that would affect respiratory and cardiovascular systems and, therefore, the IOP, all animals were restrained gently by the experimenter. No pressure was applied to the eye and neck regions during IOP measurements to avoid falsely increased IOP values (Pigatto et al., 2011). TonoVet was set on the “P” mode suitable for small rodents’ eyes according to the user manual to measure the IOP values of the right and left eyes at 5:30 (baseline) in both groups in the same lighting condition.

A single drop of 0.0015% preservative‐free tafluprost ophthalmic solution (Zioptan, Akron, Oak pharmaceutical Inc., France) was instilled in the right eyes of all animals in both groups, whereas the left eyes served as control to receive a single drop of sterile artificial teardrop.

Then, all animals in group A were exposed to 12 + 0.5 h of light from 5:30 to 18:00, whereas those in group B were exposed to 12 + 0.5 h of darkness. After 30 min, the next IOP measurement was done in groups A and B on both eyes at 6:00, and then every hour at 7:00, 8:00 and 9:00. For the rest of the 12‐h period of the experiment, the IOP measurements were performed every 3 h (at 12:00, 15:00 and 18:00) to evaluate the pattern of IOP fluctuations in both eyes. All IOP measurements and data collections were performed by a single experimenter to eliminate individual bias.

### 2.1 Statistical analysis

All the statistical analyses were performed by the SPSS statistical software (IBM, version 26.0.0.1) using repeated measure analysis of variance [ANOVA] test. Continuous variables for all groups were reported as mean ± SD, median and range because the distribution of data was not normal according to the Kolmogorov–Smirnov test.

The *p*‐values associated with the difference of the right and left eye IOP values between groups A and B (intragroup comparison) were evaluated by the Mann–Whitney test, whereas the Wilcoxon test was instrumented to compare the right and left eye IOPs in each group (intergroup comparison) at different times. All the *p*‐values equal to or less than 0.05 were considered statistically important.

## 3 RESULTS

The baseline right and left eye mean ± SD IOP values (at 5:30) were 9.0 ± 1.8 and 8.6 ± 1.2 mmHg in group A and 9.9 ± 1.9 and 9.3 ± 1.4 mmHg in group B, respectively (Table 1).

**TABLE 1:** The Mean IOP + SD (mmHg), median and range values associated with the right and left eyes of groups A and B in 16 healthy male guinea pigs during the 12 + 0.5 h of the experiment

	Group A						Group B					
	Right Eye			Left Eye			Right Eye			Left Eye		
Time	Mean IOP ± SD (mmHg)	Median	Range	Mean IOP ± SD (mmHg)	Median	Range	Mean IOP ± SD (mmHg)	Median	Range	Mean IOP ± SD (mmHg)	Median	Range
5:30 AM	9.0 ± 1.8	9.5	5.0	8.6 ± 1.2	8.5	4.0	9.9 ± 1.9	10.0	6.0	9.3 ± 1.4	9.0	5.0
6:00 AM	7.6 ± 1.8	8.5	4.0	8.1 ± 1.4	8.0	5.0	8.0 ± 1.5	8.5	5.0	8.4 ± 1.4	8.0	5.0
7:00 AM	8.2 ± 0.9	8.5	2.0	8.1 ± 0.8	8.0	2.0	8.4 ± 0.9	8.5	3.0	8.6 ± 0.9	9.0	3.0
8:00 AM	8.5 ± 1.9	9.5	5.0	8.0 ± 1.4	8.5	4.0	9.3 ± 1.7	10.0	7.0	8.7 ± 1.3	9.0	6.0
9:00 AM	9.6 ± 1.6	10	5.0	8.5 ± 1.1	9.0	3.0	9.9 ± 1.2	10.0	5.0	8.9 ± 1.2	9.0	5.0
12:00 PM	8.1 ± 1.1	8	3.0	6.9 ± 1.0	7.0	3.0	8.6 ± 1.2	9.0	4.0	7.6 ± 1.6	7.0	5.0
15:00 PM	8.4 ± 1.4	8.5	4.0	7.4 ± 1.4	7.0	3.0	9.2 ± 1.5	9.0	5.0	8.2 ± 1.3	9.0	4.0
18:00 PM	9.1 ± 1.4	9.0	5.0	8.0 ± 1.5	8.5	4.0	9.7 ± 1.4	10.0	6.0	8.8 ± 1.5	9.0	5.0

To evaluate the IOP‐lowering effect of tafluprost, the mean IOP values at each time during the experiment were analysed by repeated measure ANOVA, which is presented in Table 2. The negative values in the table are responsible for the IOP reduction in the right and left eyes in both groups.

**TABLE 2:** The Mean IOP ± SD and *p*‐values (mmHg) associated with the difference of right and left eyes mean IOP values from the baseline in groups A and B calculated by the analysis of variance (ANOVA) test in 16 healthy male guinea pigs during the 12‐h period of the experiment

	Group A				Group B			
Time	Right eye IOP reduction (mmHg)	*p*‐Value	Left eye IOP reduction (mmHg)	*p*‐Value	Right eye IOP reduction (mmHg)	*p*‐Value	Left eye IOP reduction (mmHg)	*p*‐Value
6:00 AM	−1.4 ± 1.1	0.026	−0.5 ± 1.1	<0.001	−2.5 ± 1.2	0.011	−1.2 ± 0.9	0.023
7:00 AM	−0.7 ± 1.5	<0.001	−0.5 ± 1.6	<0.001	−2.4 ± 1.6	0.017	−1.0 ± 1.8	<0.001
8:00 AM	−0.5 ± 1.1	<0.001	−0.6 ± 1.2	<0.001	−0.7 ± 1.4	<0.001	−0.6 ± 1.2	<0.001
9:00 AM	0.6 ± 0.7	0.059	−0.1 ± 1.0	<0.001	−0.7 ± 1.4	<0.001	−0.6 ± 1.1	<0.001
12:00 PM	−0.9 ± 2.5	<0.001	−1.7 ± 1.5	0.027	−1.7 ± 1.8	0.048	−1.6 ± 1.5	0.033
15:00 PM	−0.6 ± 2.2	<0.001	−1.2 ± 1.7	0.067	−0.7 ± 1.8	<0.001	−1.0 ± 1.5	<0.001
18:00 PM	0.1 ± 1.9	<0.001	−0.6 ± 2.1	<0.001	−0.6 ± 0.9	<0.001	−0.4 ± 1.6	<0.001

The right eye mean ± SD IOP values reduced significantly in group A and B at 6:00 (−1.4 ± 1.1 mmHg, *p*‐value = 0.026 and −2.5 ± 1.2 mmHg, *p*‐value = 0.011, respectively) and remained low at 7:00 (−0.7 ± 1.5 mmHg, *p*‐value < 0.001 and −2.4 ± 1.6 mmHg, *p*‐value = 0.0.17, respectively).

At 18:00, the right eye IOP reached 9.1 ± 1.4 mmHg in group A and 9.7 ± 1.4 mmHg in group B. The Mean IOP ± SD, median and range values associated with the right and left eyes of groups A and B are illustrated in Table 1.

**FIGURE 1:**
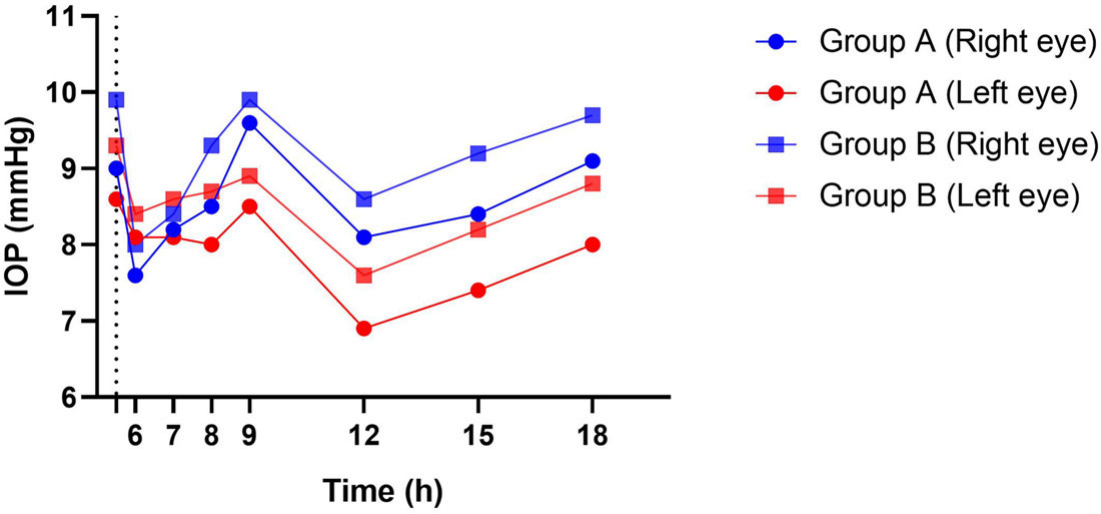
The right and left eyes mean IOP values (mmHg) and interquartile ranges associated with groups A and B illustrated by GraphPad Prism 8 software in 16 healthy male guinea pigs during the 12‐h period of the experiment

Figure 1 pictures the mean IOP fluctuations of the right and left eyes in both groups illustrated by GraphPad Prism 8 software (version 8.2.1.441).

The *p*‐values associated with the difference of the right and left eye mean IOPs between groups A and B calculated by the Mann–Whitney test are presented in Table 3, as well as those values regarding the comparison between the right and left eyes in each group by the Wilcoxon test. Although the right eye IOP values were significantly reduced in both groups post instillation of tafluprost at 6:00, this reduction was greater in group B (*p*‐value <0.001).

**TABLE 3:** The *p*‐values associated with the comparison of the right and left eyes between groups A and B (intragroup comparison) and in each group (intergroup comparison) calculated by the Mann–Whitney and Wilcoxon test, respectively, in 16 healthy male guinea pigs during the 12‐h period of the experiment

	Intragroup comparison (Mann–Whitney test)		Intergroup comparison (Wilcoxon test)	
Time	*p*‐Value (R)	*p*‐Value (L)	*p*‐Value (group A)	*p*‐Value (group B)
5:30 AM	0.065	0.050	<0.001	0.020
6:00 AM	<0.001	<0.001	<0.001	<0.001
7:00 AM	<0.001	0.083	<0.001	<0.001
8:00 AM	<0.001	0.038	<0.001	0.014
9:00 AM	<0.001	<0.001	0.024	0.058
12:00 PM	<0.001	<0.001	0.026	0.084
15:00 PM	0.021	0.038	<0.001	0.041
18:00 PM	<0.001	0.028	0.071	<0.001

There was a significant difference between the mean right and left eye IOP values in groups A (*p*‐value <0.001) and B (*p*‐value = 0.020) at 5:30 prior to the drop instillation; and also at 6:00, 7:00 and 8:00 in both groups (*p*‐value <0.05). Due to the relatively small sample size (*n* = 8), type 1 error was calculated as 0.05. The test was repeated 8 times, and the effect size was 1.5. Type 2 error was calculated according to the Web Power website as 0.94 for intragroup comparison and 0.83 for intergroup comparison (Liu, 2013).

There was a significant reduction in the IOP values of both eyes in groups A and B post instillation of tafluprost at all times because of the effect of ophthalmic solution (right eyes) and systemic absorption (left eyes), except at 9:00 for the right eye (*p*‐value = 0.059) and at 15:00 for the left eye (*p*‐value = 0.067) in group A, which is attributable to type 2 error due to small sample size.

## 4 DISCUSSION

To this day, it is the first experiment performed on guinea pigs to study the effect of tafluprost and light/darkness conditions simultaneously. According to the results of IOP measurements at baseline, the mean right eye IOP was slightly higher than the left eyes in both groups, which is attributable to the order of IOP measurements because the right eye was chosen first for all IOP recordings during the experiment (Pekmezci et al., 2011). A study by Pekmezci in 2011 demonstrated the importance of IOP measurement order, where the first measured eye always had higher IOP values, mostly due to ocular squeezing (Pekmezci et al., 2011).

Tafluprost as a topical IOP reducing ophthalmic solution was first introduced to the market in 2008 and has shown to be effective on IOP reduction in humans, dogs, monkeys and mice (Liu & Mao, 2013; Ota et al., 2007; Shokoohimand et al., 2020; Takagi et al., 2004). This ophthalmic solution was later approved in 2012 by the Food and Drug Association for use in humans for open‐angle glaucoma and ocular hypertension treatment (Ruangvaravate et al., 2020).

Previous studies on humans with normal ocular tension demonstrated that IOP was reduced significantly post instillation of tafluprost by −4.0 ± 1.7 mmHg compared to the placebo group (−1.4 ± 1.8 mmHg) after 4 weeks of therapy (*p*‐value <0.001) (Kuwayama & Komemushi, 2010). In a study conducted by Akaishi et al. (2009) in mice, IOP was significantly reduced after 1–3 h by −2.7 ± 0.6, −4.1 ± 1.3 and −5.7 ± 0.5 mmHg, respectively after tafluprost and timolol conjunctive therapy. In another study on the effect of tafluprost (single drop) on canine eyes, IOP was significantly reduced up to 6 mmHg (39% reduction) compared to the control group after 8 h (Kwak et al., 2017).

Although there was a significant difference between the right and left eye IOP values in both groups post instillation of tafluprost (except at 18:00 in group A, and also 9:00 and 12:00 in group B, Table 3), the reductions of the left eye IOP values were less than the right eye and occurred with a delay, which can be attributed to the systemic absorption of the prostaglandin analogue ophthalmic solutions.

A study by Akaishi et al. (2010) in rabbits showed that although a part of the IOP‐reducing effect of prostaglandin analogues is due to the FP receptors, the stimulation of endogenous prostaglandins in response to FP stimulation is also suggested to contribute to the systemic absorption of these drugs. In another study by Ota et al. (2007) in mice, the stimulation of prostanoid EP3 receptor by FP receptor‐mediated prostaglandin production is believed to be responsible for the IOP reduction supporting the systemic absorption thesis of tafluprost. In our study, the right eye mean IOP values differed significantly post instillation of tafluprost between groups A and B at 6:00 (*p*‐value <0.001) until the end of the experiment at 18:00 (*p*‐value <0.001). Moreover, the IOP reductions associated with the right and left eyes were significant at all times in group B (*p*‐value <0.05) and were greater in amount in comparison with group A due to the effect of darkness on IOP.

IOP fluctuations during the day are influenced by several biological factors such as circadian rhythms first introduced by Halberg in 1959, which in humans are a function of suprachiasmatic nuclei located in the anterior hypothalamus (Halberg, 1959; Moore et al., 2002; Reiss et al., 1984; Smolensky & Haus, 2001). Although the activity of the internal pacemaker can happen in the absence of external stimuli, many environmental factors contribute to its function, most importantly, the daily cycles of light and darkness (Buijs & Kalsbeek, 2001; Cahill & Menaker, 1989).

Although there have been several studies on the diurnal rhythms of IOP in different animal species, there are some significant variations among them, indicating a direct relationship between higher IOP values and activity/awakening phases (Del Sole et al., 2007). A study on the effect of circadian rhythm on daily IOP fluctuations in rabbits (12 h light/12 h darkness) revealed that IOP values increased by 10 mmHg (Alcon Applanation Pneumography) with the onset of darkness, whereas decreased by the same amount with the onset of light (Rowland et al., 1981). A similar study on rats evaluated the diurnal pattern of IOP fluctuations, in which IOP values, measured by TonoPen XL tonometer, first decreased and then increased in light (19.3 ± 1.9 mmHg) and dark (31.3 ± 1.3 mmHg) conditions, respectively (Moore et al., 1996). With regard to previous literature, in nocturnal species such as cats, the maximum IOP values were recorded at night (Del Sole et al., 2007), whereas in both rhesus macaques and beagle dogs, IOP was highest in the early morning (Bito et al., 1979; Chen et al., 1980).

In support, the daily IOP fluctuations (12:12 light/dark) of the guinea pigs (TonoLab, Colonial Medical Supply, NH, USA) have been investigated by Ostrin et al. in 2016 to have reached their peak at 9:00 in the morning (25.8 ± 2.5 mmHg) and then decreased to 20.1 ± 0.9 mmHg at 9:00 pm thorough the day (Ostrin & Wildsoet, 2016). However, the IOP values of guinea pigs were lowest during the morning (3.68 mmHg) at 7:00 and highest during the night (8.12 mmHg) at 23:00 in another study during a 12:12‐h light/dark photocycle measured by rebound tonometry (TonoVet, iCare; Helsinki, Finland) (Ansari‐Mood et al., 2016).

Prostaglandin analogue ophthalmic solutions, such as tafluprost, are considered safe and convenient when applied once daily. Even though several local side effects (conjunctival hyperemia, mild irritation of the eye and pigmentation of eyelid, periocular skin and iris) have been reported in prolonged use of tafluprost in less than 7.7% of patients (Feldman, 2003; Honrubia et al., 2009; Kuwayama & Nomura, 2014; Papadia et al., 2011), no local side effects were observed in the guinea pigs post instillation of a single drop of tafluprost during the 12 + 0.5 h of the experiment.

## 5 CONCLUSIONS

The results of the current study demonstrate that a variation in the IOP values of the guinea pigs post instillation of tafluprost is present between the two groups, which is attributable to the carefully monitored conditions of light and darkness. Therefore, IOP fluctuations during the day and night are important parameters to be considered in ocular hypertension treatment.

## AUTHOR CONTRIBUTIONS

*Data curation; formal analysis; funding acquisition; investigation; resources; writing – original draft; writing – review and editing*: Arghavan Armin. *Conceptualization; methodology; supervision; validation; visualization; writing – review and editing*: Farnoosh Arfaee. *Supervision; validation*: Saeed Ozmaie. *Supervision; validation*: Ahmad Asghari.

## CONFLICT OF INTERESTS

The authors declare that they have no known competing financial interests or personal relationships that could have appeared to influence the work reported in this paper. No financial or conflict of interest that might have biased the work have been declared.

### ETHICS STATEMENT

All guidelines regarding the work with laboratory animals have been observed during the experiment and animals handling. The Ethics Committee of the Science and Research Branch of Islamic Azad University approved this study for the Use of Animals in Ophthalmic and Vision Research (IR.IAU.SRB.REC.1399.078).

### PEER REVIEW

The peer review history for this article is available at https://publons.com/publon/10.1002/vms3.1082

## Data Availability

The data that support the findings of this study are available on request from the corresponding author. The data are not publicly available due to privacy or ethical restrictions.
